# Discrepancy between radiological and pathological size of renal masses

**DOI:** 10.1186/1471-2490-11-2

**Published:** 2011-02-22

**Authors:** Nicola N Jeffery, Norbert Douek, Ding Y Guo, Manish I Patel

**Affiliations:** 1 Royal Prince Alfred Hospital, Missenden Rd, Camperdown, NSW, 2050, Australia; 2 Campbelltown Hospital, Therry Rd, Campbelltown, NSW, 2560, Australia; 3 Concord Hospital, Hospital Rd, Concord, NSW, 2139, Australia; 4 Discipline of Surgery, University of Sydney, Westmead, NSW, 2145, Australia

## Abstract

**Background:**

Tumor size is a critical variable in staging for renal cell carcinoma. Clinicians rely on radiological estimates of pathological tumor size to guide patient counseling regarding prognosis, choice of treatment strategy and entry into clinical trials. If there is a discrepancy between radiological and pathological measurements of renal tumor size, this could have implications for clinical practice. Our study aimed to compare the radiological size of solid renal tumors on computed tomography (CT) to the pathological size in an Australian population.

**Methods:**

We identified 157 patients in the Westmead Renal Tumor Database, for whom data was available for both radiological tumor size on CT and pathological tumor size. The paired Student's *t*-test was used to compare the mean radiological tumor size and the mean pathological tumor size. Statistical significance was defined as *P *< 0.05. We also identified all cases in which post-operative down-staging or up-staging occurred due to discrepancy between radiological and pathological tumor sizes. Additionally, we examined the relationship between Fuhrman grade and radiological tumor size and pathological T stage.

**Results:**

Overall, the mean radiological tumor size on CT was 58.3 mm and the mean pathological size was 55.2 mm. On average, CT overestimated pathological size by 3.1 mm (*P *= 0.012). CT overestimated pathological tumor size in 92 (58.6%) patients, underestimated in 44 (28.0%) patients and equaled pathological size in 21 (31.4%) patients. Among the 122 patients with pT1 or pT2 tumors, there was a discrepancy between clinical and pathological staging in 35 (29%) patients. Of these, 21 (17%) patients were down-staged post-operatively and 14 (11.5%) were up-staged. Fuhrman grade correlated positively with radiological tumor size (*P *= 0.039) and pathological tumor stage (*P *= 0.003).

**Conclusions:**

There was a statistically significant but small difference (3.1 mm) between mean radiological and mean pathological tumor size, but this is of uncertain clinical significance. For some patients, the difference leads to a discrepancy between clinical and pathological staging, which may have implications for pre-operative patient counseling regarding prognosis and management.

## Background

Tumor size is an important prognostic indicator for renal cell carcinoma (RCC), and is thus a critical variable in staging systems and a key factor when deciding upon treatment strategy.

The 2009 TNM staging system for RCC stratifies tumors limited to the kidney by their size alone (T1a ≤4 cm; T1b > 4 cm but ≤7 cm; T2a > 7 cm but ≤10 cm; T2b > 10 cm)[1]. Available prognostic nomograms also incorporate tumor size[2-5].

Renal tumor size also guides clinicians in recommending radical nephrectomy (RN), partial nephrectomy (PN), ablative techniques or active surveillance as the management of choice. PN is the standard approach for T1a (≤4 cm) renal tumors, achieving equivalent oncological efficacy to RN[6], while preserving renal function[7] and protecting from non-cancer related mortality[8,9]. Several studies support PN for all amenable T1b tumors (> 4 cm but ≤7 cm) [10-14]. The growing acceptance of PN as an option for T1b tumors is reflected in current American and European guidelines[15,16]. RN remains the therapy of choice for T2 tumors (> 7 cm) [16,17]. Although recent studies have demonstrated the feasibility of PN for carefully selected patients with T2 tumors in experienced centers[18,19], it is uncertain whether these results can be extrapolated to all institutions. For high-risk surgical candidates with small renal tumors, there is intermediate-term data to support minimally invasive ablative techniques such as cryoablation and radiofrequency ablation (RFA) [20]. There is a relationship between tumor size and local recurrence after ablation[20], and a tumor size threshold of 3.5 cm has been proposed for such techniques[17]. In patients with limited life expectancy, active surveillance of small renal masses has been advocated as a viable option, provided that tumor size is less than 3 cm[21].

Most studies report patient outcomes following surgical intervention for RCC according to the pathological size of the tumor, rather than the radiological size on CT[2-5,22,23]. Indeed, the studies that have defined a tumor size threshold for partial nephrectomy are all based on pathological size[6,7,10-14].

Preoperatively, clinicians must rely on radiological estimates of pathological tumor size to guide patient counseling regarding prognosis and management. For example, at institutions employing a size threshold for PN, patients will be offered or denied PN based on tumor size on CT. If there is a discrepancy between radiological size on CT and pathological size, this may have implications for clinical practice.

For patients undergoing ablative techniques, pathological tumor size cannot be determined. Therefore, studies report the outcome of ablative techniques according to radiological tumor size[20]. If a discrepancy between radiological and pathological tumor size exists, it may be difficult to meaningfully compare these studies with the established evidence for nephrectomy, which is reported according to pathological size.

A number of studies have examined the relationship between CT size and pathological size of renal tumors[24-36]. Most of these studies found that, on average, CT overestimated pathological tumor size, although this reached statistical significance in only three studies[28,33,35]. Authors have disagreed on the clinical significance of these findings. Only two of these studies comprehensively reported instances in which disagreement between CT and pathological size led to discordance between clinical and pathological stage[26,30]. To our knowledge, there has been no such study performed on an Australian population. A recent study has demonstrated different trends in stage migration in an Australian RCC cohort compared with populations in the USA[37]. Therefore, international findings are not necessarily applicable to the Australian population and there is a need for Australian data to be reported.

The aim of our study was to compare the radiological size of RCC on CT to the pathological size in a contemporary Australian population. We also aimed to identify patients who were up-staged or down-staged due to discrepancy between CT and pathological size.

## Methods

The Westmead Renal Tumor Database contains 547 patients whose tumors were removed by radical or partial nephrectomy from 1994 to 2007. Data collection and analysis was approved by the hospital ethics committee and complies with the Declaration of Helsinki.

We retrospectively reviewed the database and identified 157 patients for whom accurate data was available for both radiological and pathological tumor size. Radiological tumor size was defined as the largest transverse diameter in the axial plane on CT scan, as measured by the reporting radiologist. The CT protocol entailed pre-contrast images and images in the arterial, corticomedullary (venous) and excretory phases. Tumor size was measured in the phase in which the tumor margins were most obvious. Coronal and sagittal reconstruction images were available, but the radiological tumor size was always measured in the axial plane. Pathological tumor size was defined as the largest transverse diameter, as measured by the pathologist at examination of the surgical specimen prior to formalin fixation. There were 4 patients with multifocal tumors. For these patients, we included the data for their largest tumor in our analysis. According to radiological size, tumors were grouped by 1 cm size intervals and by clinically relevant size intervals (≤4 cm; >4 cm but ≤7 cm; >7 cm but ≤10 cm; >10 cm).

We extracted demographic data for all patients from the database, including age, sex, year of operation, type of procedure (open or laparoscopic, radical or partial nephrectomy), tumor histology (conventional, papillary, chromophobe, other), Fuhrman grade, and clinical and pathological tumor stage (according to 2009 TNM staging system).

The paired Student's *t*-test was used to compare the mean radiological tumor size and the mean pathological tumor size. Statistical significance was defined as *P *< 0.05. Data analysis was performed using SPSS, version 15.0. We also compared mean radiological and mean pathological size for tumors grouped by histological subtype, by type of procedure, by 1 cm size intervals and by clinically relevant size intervals (≤4 cm; >4 cm but ≤7 cm; >7 cm but ≤10 cm; >10 cm).

For patients with pT1 and pT2 tumors, the radiological and pathological tumor sizes were compared to identify all cases of post-operative down-staging or up-staging. We calculated the number and percentage of patients for whom a difference between radiological and pathological tumor sizes accounted for discrepancy between clinical and pathological tumor stage.

We also examined the relationship between Fuhrman grade and CT tumor size (grouped into clinically relevant size intervals) and pathological T stage using a chi-square test. Of our cohort of 157 patients, 7 were excluded from this analysis because they did not have a Fuhrman grade recorded in the database. For the analysis we grouped tumors into low-grade (Fuhrman 1 or 2) and high-grade (Fuhrman 3 or 4).

## Results

A total of 157 patients were identified, among whom there were 51 (32.5%) women and 106 men (67.5%). The mean (range) patient age was 63.3 (34-100) years. The patients underwent surgery between 1998 and 2007. There were 18 (11.5%) patients treated with partial nephrectomy (10 laparoscopic and 8 open procedures), and 139 (88.5%) treated with radical nephrectomy (100 laparoscopic and 39 open). The histological tumor subtype was conventional in 126 (80.3%) patients, papillary in 16 (10.2%), chromophobe in 11 (7.0%) and other in 4 (2.5%). The pathological tumor stage (according to the 2009 TNM staging system) was T1a in 58 (36.9%) patients, T1b in 41 (26.1%), T2a in 18 (11.5%), T2b in 5 (3.2%), T3a in 30 (19.1%), T3b in 2 (1.3%), T3c in 2 (1.3%) and T4 in 1 (0.6%). Demographic data for our study population is summarized in *Table *1.

**Table 1:** Demographic data for 157 patients

Feature	*N *or Mean (%)	
**Age **(years)	63.3	(34 - 100)
**Sex**		
Male	51	(32.5)
Female	106	(67.5)
**Year of Procedure**		
1998	2	(1.3)
1999	2	(1.3)
2000	6	(3.8)
2001	10	(6.4)
2002	18	(11.5)
2003	16	(10.2)
2004	10	(6.4)
2005	21	(13.4)
2006	34	(21.7)
2007	38	(24.2)
**Type of Procedure**		
Laparoscopic PN†	10	(6.4)
Laparoscopic RN‡	100	(63.7)
Open PN	8	(5.1)
Open RN	39	(24.8)
**Histological subtype**		
Conventional	126	(80.3)
Papillary	16	(10.2)
Chromophobe	11	(7.0)
Other	4	(2.5)
Mixed	2	
Neuroendocrine	1	
Unclassified	1	
Pathological T stage		
pT1a	58	(36.9)
pT1b	41	(26.1)
pT2a	18	(11.5)
pT2b	5	(3.2)
pT3a	30	(19.1)
pT3b	2	(1.3)
pT3c	2	(1.3)
pT4	1	(0.6)

† PN - Partial nephrectomy

‡ RN - Radical nephrectomy

**Figure 1:**
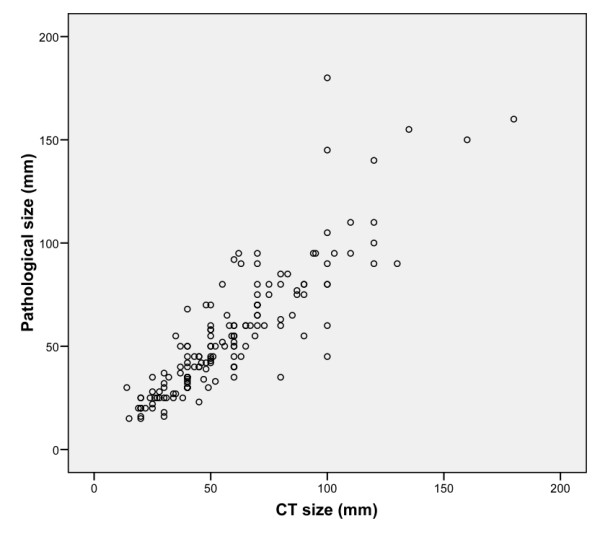
**Scatter plot of pathological tumor size against radiological tumor size**. Please see attached image file.

A scatter plot of pathological tumor size against radiological tumor size is shown in *Figure *1. Overall, the mean radiological tumor size on CT was 58.3 mm (SD 29.2 mm) and the mean pathological size was 55.2 mm (SD 30.5 mm). On average, CT overestimated pathological size by 3.1 mm (95% CI: 0.7 to 5.5 mm, *P *= 0.012). CT overestimated pathological tumor size in 92 (58.6%) patients, underestimated in 44 (28.0%) patients and equaled pathological size in 21 (31.4%) patients.

Among the 122 patients with pT1 or pT2 tumors, there was a discrepancy between clinical and pathological staging in 35 (29%) patients. Of these, 21 (17%) patients were down-staged post-operatively and 14 (11.5%) were up-staged. This data is summarized in *Table *2.

**Table 2:** Discrepancy between clinical and pathological stage in 122 pT1 and pT2 tumors.

	Clinical stage (n)	Pathological stage (n)	Downstaged (n)	Upstaged (n)
T1a	52	58	-	6 cT1a to pT1b
T1b	48	41	12 cT1b to pT1a	6 cT1b to pT2a
T2a	15	18	4 cT2a to pT1b 1 cT2a to pT1a	2 cT2a to pT2b
T2b	7	5	4 cT2b to pT2a	-
Total	122	122	21 (17%)	14 (11.5%)

*Table *3 shows the mean radiological and pathological tumor sizes divided into 10 mm size intervals by radiological size. Mean radiological size was greater than mean pathological size for all size intervals, except for the 50 - 59 mm and 70 - 79 mm categories. This only reached statistical significance for tumors in the 80 - 89 mm category, for which mean radiological size was 13 mm larger than mean pathological size (95% CI: 1.26 to 24.74 mm, *P *= 0.034).

**Table 3:** Mean radiological and pathological tumor size (mm) divided into 10 mm size intervals by radiological size.

**CT size (mm) **†	*N*	**Mean CT size (SD) **‡	Mean pathological size (SD)	**Mean difference (95% CI) **§	*P *value
10 to 19	3	16.00 (2.65)	21.67 (7.64)	5.67 (-16.60 to 27.90)	0.388
20 to 29	17	23.24 (3.11)	23.18 (4.85)	0.59 (-1.99 to 2.10)	0.952
30 to 39	16	33.13 (3.07)	31.50 (10.56)	1.63 (-3.45 to 6.70)	0.506
40 to 49	32	42.41 (3.19)	39.19 (10.12)	3.22 (-0.42 to 6.85)	0.081
50 to 59	20	52.25 (3.14)	53.25 (2.40)	-1.00 (-5.76 to 3.76)	0.665
60 to 69	22	61.77 (2.74)	56.55 (16.37)	5.23 (-1.93 to 12.39)	0.144
70 to 79	13	71.00 (1.96)	73.46 (10.68)	-2.46 (-9.02 to 4.10)	0.430
80 to 89	9	82.44 (3.13)	69.44 (15.89)	13.00 (1.26 to 24.74)	0.034
90 to 99	6	91.50 (2.35)	80.00 (14.83)	11.50 (-2.24 to 25.24)	0.084
≥100	19	116.21 (22.29)	109.47 (36.09)	6.74 (-8.38 to 21.85)	0.361

† mm = millimetres

‡ SD = standard deviation

§ CI = confidence interval

*Table *4 shows the mean radiological and pathological tumor sizes separated into clinically relevant size intervals, corresponding to T1a (≤4 cm), T1b (>4 cm but ≤7 cm), T2a (>7 cm but ≤10 cm) and T2b (>10 cm) stages. For all three groups, mean radiological size was greater than mean pathological size but the difference did not achieve statistical significance.

**Table 4:** Mean radiological and pathological tumor size (mm) divided into clinically relevant size intervals by radiological size.

CT size (mm)	*N*	Mean CT size (SD)	Mean pathological size (SD)	Mean difference (95% CI)	*P *value
≤40	55	31.51 (8.17)	30.58 (10.59)	0.93 (-1.30 to 3.15)	0.407
> 40 but ≤70	65	56.94 (8.50)	55.14 (16.08)	1.80 (-1.43 to 5.03)	0.269
> 70 but ≤100	26	89.00 (9.26)	80.96 (29.05)	8.04 (-2.66 to 18.73)	0.134
> 100	11	128.00 (23.04)	117.73 (27.78)	10.27 (-2.17 to 22.71)	0.096

† SD = standard deviation

‡ CI = confidence interval

*Table *5 shows the mean radiological and pathological tumor sizes for the different histological sub-types. For conventional RCC, CT overestimated pathological size by an average of 3.8 mm (95% CI 1.25 to 6.39 mm, *P *= 0.004). There was no statistically significant difference for the other histological subtypes.

**Table 5:** Mean radiological and pathological tumor size (mm) by histological subtype

Histology	*N*	**Mean CT size (SD) **†	Mean pathological size (SD)	**Mean difference (95% CI) **‡	*P *value
Conventional	126	60.37 (29.79)	56.56 (31.06)	3.82 (1.25 to 6.39)	0.004
Papillary	16	43.44 (22.82)	43.75 (20.12)	-0.31 (-6.46 to 5.84)	0.915
Chromophobe	11	52.82 (29.12)	53.00 (38.21)	-0.18 (-16.94 to 16.57)	0.981
Other	4	68.25 (19.97)	64.25 (18.95)	4.00 (-35.21 to 43.21)	0.767

† SD = standard deviation

‡ CI = confidence interval

*Table *6 shows the mean radiological and pathological tumor sizes stratified by type of procedure. For tumors removed by radical nephrectomy, the mean radiological size was 3.4 mm larger than the mean pathological size (95% CI: 0.71 to 6.02 mm, *P = *0.013). There was no statistically significant difference detected for tumors removed by partial nephrectomy.

**Table 6:** Mean radiological and pathological tumor size (mm) stratified according to type of procedure

Type of surgery	*N*	**Mean CT size (SD) **†	Mean pathological size (SD)	**Mean difference (95% CI) **‡	*P *value
Partial nephrectomy	18	33.33 (14.21)	32.11 (13.11)	1.22 (-4.61 to 7.06)	0.664
Radical nephrectomy	139	61.55 (29.13)	58.19 (30.84)	3.37 (0.71 to 6.02)	0.013

† SD = standard deviation

‡ CI = confidence interval

*Table *7 shows radiological tumor size (grouped into clinically relevant size intervals) distributed according to Fuhrman grade. High-grade disease (Fuhrman 3 or 4) was more common in larger tumors (≤4 cm vs >4 cm but ≤7 cm vs >7 cm; *P *= 0.039). The prevalence of high-grade disease was 24.5%, 31.1% and 50.0% for tumors ≤4 cm, >4 cm but ≤7 cm, >7 cm respectively. *Table *8 shows pathological T stage (grouped into T1a, T1b and ≥ T2) distributed according to Fuhrman grade. There was a statistically significant positive correlation between Fuhrman grade and tumor stage (*P *= 0.003).

**Table 7:** Radiological tumor size (mm) distributed according to Fuhrman grade.

		Fuhrman Grade					
		I	II	III	IV	N/A†	Total
**Radiological tumor size (mm)**	≤ 40	11	29	13	0	2	55
	>40 but ≤ 70	5	37	18	1	4	65
	> 70	4	14	13	5	1	37
**Total**		20	80	44	6	7	157

† N/A = Not available.

**Table 8:** Pathological T stage distributed according to Fuhrman grade.

		Fuhrman Grade					
		I	II	III	IV	N/A†	Total
**Pathological T stage**	T1a	11	34	12	0	1	58
	T1b	5	22	9	1	4	41
	≥T2	4	24	23	5	2	58
**Total**		20	80	44	6	7	157

† N/A = Not available.

## Discussion

Tumor size is an important prognostic indicator for RCC. Outcome of nephrectomy has been studied according to pathological tumor size. Pre-operatively, we must rely upon CT estimates of pathological tumor size to guide counseling regarding prognosis and choice of treatment modality. Furthermore, ablative techniques for renal tumors do not provide specimens for pathological assessment of tumor size. When comparing emerging ablative techniques to the benchmark of nephrectomy, we are comparing data based on pathological tumor size to data based on CT size. Therefore, it is important to understand the relationship between radiological tumor size and pathological tumor size, and to understand how any difference between the two measurements affects the accuracy of clinical staging.

Our study of a contemporary Australian cohort found that overall CT overestimated pathological tumor size by a statistically significant but small amount (3.1 mm). This observation is consistent with the findings of previous studies. The findings of recent papers comparing mean radiological and mean pathological renal tumors sizes are summarized in *Table *9. Kurta et al[28] reported on the largest series (*N *= 521), and found that mean radiological tumor size was larger than mean pathological tumor size by 1 mm. Similarly, CT was found to overestimate pathological tumor size overall by 6.3 mm in a study by Herr[35], and by 10.0 mm in a paper by Irani et al[33]. Schlomer et al[31] found no statistically significant difference overall, but found that CT overestimated pathological size for pT1a tumors by 3.9 mm and for lesions 40 to 50 mm by 8.7 mm. Similarly, Lee et al[24] found a statistically significant overestimation of pathological tumor size by CT for tumors in the 40 to 50 mm range only, by an average of 2 mm. Choi et al[25] found that CT tumor size was on average larger than pathological size for smaller tumors only (<6 cm or T1). In several other series, mean radiological tumor size was greater than mean pathological size, but the difference did not reach statistical significance[27,29,30,32,34,36]. Only one study reported an underestimation of pathological tumor size by CT overall, and this achieved statistical significance for T1a tumors only[26].

**Table 9:** Summary of previous studies comparing mean radiological and mean pathological renal tumor sizes.

Author	Year	N	**Mean CT size (mm) **‡	Mean Path size (mm)	Mean δ (mm)	P value
Herr[35]	1999	50	N/A	N/A	6.3	**0.001**
Herr et al[34]	2001	87	34	27	7.4	N/A
Irani[33] †	2001	100	70.0	60.0	10.0	**0.005**
Yaycioglu[32]	2002	291	54	53	1.0	0.17
Schlomer[31]	2006	133	44.7	41.4	3.3	0.35
Kanofsky[30]	2006	198	51.1	49.2	1.9	N/A
Mistry[29]	2008	106	49.9	47.4	2.5	0.70
Kurta[28]	2008	521	47.9	46.9	1.0	**0.02**
Alicioglu[27]	2009	35	75.0	62.5	12.5	0.452
Ates[26]	2010	86	63.3	64.3	-1.0	0.342
Choi[25]	2010	175	49.8	45.5	4.3	0.152
Lee[24]	2010	467	45.6	44.9	0.7	0.399
Jeffery	2010	157	58.3	55.2	3.1	**0.012**

† Used median CT size and median pathological size.

‡ mm = millimetres

Analysis by histological subtype in our series showed a statistically significant difference for conventional RCC only, with CT overestimating pathological size by an average of 3.8 mm. The small number of papillary (*N *= 18) and chromophobe (*N *= 11) tumors included in our study meant we were unlikely to detect a statistically significant difference. Several studies have shown that CT size is greater than pathological size on average for conventional RCC, and smaller than pathological size on average for papillary RCC[24,28,32]. Kurta et al[28] found that CT overestimated pathological tumor size by 2.3 mm for conventional RCC and underestimated pathological tumor size by 5.4 mm for papillary RCC. Similarly, Lee et al[24] found that CT size was 1.4 mm greater than pathological size on average for conventional RCC, and 5.3 mm smaller for papillary RCC. In contrast, Herr[34] found that pathological size was overestimated on CT for all histological subtypes, and that the overestimation was significantly greater for conventional RCC compared to other subtypes (9.7 mm versus 3.9 mm). Similarly, Choi et al[25] demonstrated that mean radiological tumor size was larger than mean pathological tumor size for all histological subtypes, but there was no significant difference between groups.

The discrepancy between clinical and pathological tumor size has been attributed to decreased tumor vascularity after excision, leading to a diminished size post-operatively[34]. This effect is probably more pronounced for clear cell carcinomas because they typically have a richer vascular network than other histological subtypes. Yaycioglu et al[32] postulated that certain radiological and pathological features might influence the accuracy of tumor size measurement by CT. These features included: concomitant pyelonephritis, presence of hemorrhage or hematoma, cystic tumor or adjacent cysts, dilatation of adjacent renal calyces and invasion of the collecting system. The same study found that tumor invasion of perinephric tissues impacted upon the accuracy of CT. For these tumors, CT more frequently underestimated pathological size when compared to tumors confined to the kidney. Ates et al[26] demonstrated less accurate CT measurement of tumor size for locally invasive tumors. It may be more difficult to delineate the radiographic margin of invasive tumors on CT, leading to disagreement between radiological and pathological tumor sizes. Ates et al[26] also found more accurate measurement of tumors size on CT for exophytic lesions. Herr[35] found that CT more closely approximated pathological tumor size for upper pole tumors, but other studies have failed to confirm this finding[24,32,33]. Additionally, in our study the radiological and pathological tumor sizes were not necessarily measured in the same geometric plane and this could contribute to the discrepancy between the two measurements. The largest tumor diameter on CT was measured in the axial plane, and this did not always correspond to the plane in which the largest diameter was measured at pathological exam. Formalin fixation is known to cause tumor shrinkage[38], but in our series the pathological specimens were examined prior to fixation.

Inaccurate CT estimation of pathological tumor size led to discordance between clinical and pathological stage in over one quarter of tumors limited to the kidney in our study (pT1, pT2). Of these, 21 (17%) patients were down-staged and 14 (11.5%) up-staged post-operatively. There is limited published data on the impact that disagreement between radiological and pathological tumor sizes may have on staging discrepancies. Kanofsky et al[30] reported on a series of 198 renal cell carcinomas and identified 21 patients for whom disagreement between CT and pathological tumor size led to discrepancy between clinical and pathological tumor stage. Of these, 15 patients were down-staged and 6 up-staged post-operatively. Ates et al[26] found that differences between radiological and pathological measurements led to staging discrepancies in 19 of 86 patients, with 6 patients being down-staged and 13 patients being up-staged post-operatively. Kurta et al[28] and Lee et al[24] only reported cases of post-operative down-staging. Kurta et al demonstrated that among 258 patients with CT tumor size greater than 4 cm, 30 (11.6%) had a pathological size of less than 4 cm. Among 92 patients with CT tumor size greater than 7 cm, 7 (7.6%) had a pathological size of less than 7 cm. Lee et al demonstrated similar results. Of the 141 patients with CT tumor size between 4 cm and 7 cm, 17 (12.1%) had a pathological size less than 4 cm. Of the 87 patients with CT tumor size greater than 7 cm, 8 (9.2%) had a pathological size of less than 7 cm.

For these patients, pre-operative counseling regarding prognosis and management would have been based on a clinical tumor stage that was ultimately down-staged or up-staged based on pathological tumor size. Thus, although the magnitude of the mean difference between radiological and pathological tumor sizes is only 3.1 mm, there are cases where the discrepancy may impact upon clinical management.

Authors disagree about the clinical implications of the small but statistically significant difference between CT and pathological tumor size. Some studies conclude that CT adequately approximates pathological tumor size[24,26,32,34], and that any discrepancy between the two measurements has minimal impact on patient management[28]. Other authors point out that overestimation of pathological size on CT could affect selection of patients for elective PN[29,31,33,34]. PN is the standard of care for T1a tumors (≤4 cm) [17]. Mistry et al[29] report that 5 (5%) of their patients who were not offered elective PN based on a CT tumor size > 40 mm, had a pathological size ≤4 cm. Likewise, 3 patients out of 100 included in the study by Irani et al[33] were ineligible for elective PN based on CT size > 40 mm, but had a pathological size ≤4 cm. However, with the growing impetus to use PN for all amenable T1 tumors[15,16], tumor size is becoming less important for determining patient eligibility for PN. Several authors argue that the decision to perform elective PN should be based on technical feasibility and patient preference rather than a rigid tumor size cut-off[12,13,18,19,39].

The discrepancy between radiological and pathological tumor size could have implications for the use of ablative techniques and active surveillance for RCC. These approaches produce no specimen for pathological assessment, and so we must rely upon CT estimates of tumor size to guide management. Decision-making under these circumstances is aided by the small number of studies that report tumor prognosis according to radiological tumor size. Kanao et al[40] have recently developed a preoperative prognostic nomogram based on clinical staging to predict survival after nephrectomy. Raj et al[41] have also developed a preoperative nomogram to predict the development of metastases after nephrectomy. Such prognostic data based on clinical information can be used as a benchmark against which the oncological outcome of ablative techniques can be compared.

Our finding of a positive correlation between Fuhrman grade and tumor size supports previous observations. Thompson et al[42] (*N = *1523) and Frank et al[43] (*N = *2559) both demonstrated that larger tumors were more likely to harbor high-grade disease, with each 1 cm increase in tumor size carrying a 25 - 32% increased risk of high-grade disease (Fuhrman 3 or 4). Analysis of tumors grouped according to various tumor size breakpoints (3 cm[44], 4 cm[45], 5 cm[46]) has also shown a higher prevalence of high-grade disease in the larger size groups. In contrast, Klatte et al classified tumors by an 11 cm breakpoint and found that Fuhrman grade was similar in the two groups[47]. Our finding that Fuhrman grade correlated with tumor stage is also consistent with findings from other studies[48,49]. The relationship between tumor size and Fuhrman grade has implications for patient counseling and management, particularly if electing active surveillance.

Our study has several shortcomings. It is a retrospective single institution analysis. The small numbers of papillary and chromophobe histological subtypes, and the small number of patients treated with partial nephrectomy were inadequately powered to detect a difference. Likewise, when categorized into 1 cm size intervals, several groups had insufficient numbers to detect a difference. There was no record of when the pre-operative CT was performed, and so we could not standardize the interval between imaging and surgery. Furthermore, there was no uniform protocol for measurement of CT tumor size and pathological tumor size. There was no centralized review of measurements by a single radiologist or pathologist.

A follow-up prospective multi-centre study with larger numbers and a uniform protocol for tumor measurement should be performed to further elucidate the relationship between CT and pathological tumor size. There is also a need for studies examining the correlation between clinical and pathological staging for RCC. Studies that report prognosis according to radiological rather than pathological tumor size would guide us in making treatment decisions based on clinical tumor size. The development and validation of pre-operative prognostic nomograms would also aid decision-making.

## Conclusions

There was a statistically significant but small overestimation (3.1 mm) of pathological size by CT overall, but this is of uncertain clinical significance. For some patients, the difference leads to a discrepancy between clinical and pathological staging, which may have implications for pre-operative patient counseling regarding prognosis and choice of treatment strategy.

## Competing interests

The authors declare that they have no competing interests.

## Authors' contributions

NJ drafted the manuscript. ND and DG were responsible for creating and maintaining the Westmead Renal Tumor database. MP conceived the idea of the study and revised the manuscript. All authors have read and approved the final manuscript.

## Pre-publication history

The pre-publication history for this paper can be accessed here:

http://www.biomedcentral.com/1471-2490/11/2/prepub
